# A ventilation intervention study in classrooms to improve indoor air quality: the FRESH study

**DOI:** 10.1186/1476-069X-12-110

**Published:** 2013-12-17

**Authors:** Jeannette TM Rosbach, Machiel Vonk, Frans Duijm, Jan T van Ginkel, Ulrike Gehring, Bert Brunekreef

**Affiliations:** 1 Institute for Risk Assessment Sciences, Utrecht University, P.O. Box 80178, 3508 TD, Utrecht, The Netherlands; 2 Department of Environmental Health, Municipal Health Services Groningen, P.O. Box 584, 9700 AN, Groningen, The Netherlands; 3 Department of Environmental Health, Municipal Health Services IJsselland, P.O. Box 1453, 8001 BL, Zwolle, The Netherlands; 4 Julius Center for Health Sciences and Primary Care, University Medical Center Utrecht, P.O. Box 85500, 3508 GA, Utrecht, The Netherlands

**Keywords:** Ventilation, Schools, Carbon dioxide, Indoor air quality, Intervention

## Abstract

**Background:**

Classroom ventilation rates often do not meet building standards, although it is considered to be important to improve indoor air quality. Poor indoor air quality is thought to influence both children’s health and performance. Poor ventilation in The Netherlands most often occurs in the heating season. To improve classroom ventilation a tailor made mechanical ventilation device was developed to improve outdoor air supply. This paper studies the effect of this intervention.

**Methods:**

The FRESH study (Forced-ventilation Related Environmental School Health) was designed to investigate the effect of a CO_2_ controlled mechanical ventilation intervention on classroom CO_2_ levels using a longitudinal cross-over design. Target CO_2_ concentrations were 800 and 1200 parts per million (ppm), respectively. The study included 18 classrooms from 17 schools from the north-eastern part of The Netherlands, 12 experimental classrooms and 6 control classrooms. Data on indoor levels of CO_2_, temperature and relative humidity were collected during three consecutive weeks per school during the heating seasons of 2010–2012. Associations between the intervention and weekly average indoor CO_2_ levels, classroom temperature and relative humidity were assessed by means of mixed models with random school-effects.

**Results:**

At baseline, mean CO_2_ concentration for all schools was 1335 ppm (range: 763–2000 ppm). The intervention was able to significantly decrease CO_2_ levels in the intervention classrooms (F (2,10) = 17.59, p < 0.001), with a mean decrease of 491 ppm. With the target set at 800 ppm, mean CO_2_ was 841 ppm (range: 743–925 ppm); with the target set at 1200 ppm, mean CO_2_ was 975 ppm (range: 887–1077 ppm).

**Conclusions:**

Although the device was not capable of precisely achieving the two predefined levels of CO_2_, our study showed that classroom CO_2_ levels can be reduced by intervening on classroom ventilation using a CO_2_ controlled mechanical ventilation system.

## Background

Children spend much of their time in schools; it is the indoor environment where they spend most of their time besides in their home. It is therefore important that schools have a good indoor air quality (IAQ). Classroom ventilation was already recognised as an important determinant of indoor air quality in the beginning of the 20^th^ century [1]; however, even recent studies showed that classroom ventilation rates do not meet building standards. Two studies performed in The Netherlands in 2007 showed that more than 80% of the schools exceeded CO_2_ levels of 1200 parts per million (ppm) during classroom occupation [2,3], which in The Netherlands is the advised maximum CO_2_ concentration for classrooms [4,5].

Poor IAQ has found to be associated with a negative impact on health [6,7]. However, these reviews mainly focussed on office buildings and their occupants. Daisey et al. [8] reviewed the literature published until 1999 with a specific focus on schools. With respect to ventilation, most studies merely investigated the amount of ventilation and conclude that ventilation is inadequate in many classrooms, which may possibly lead to health related symptoms. As of 1999, Daisy et al. [8] found two studies that specifically looked at the relationship between ventilation and the prevalence of health related symptoms. However, the results of these two studies were inconsistent and thus the authors stress the need of more studies looking into the relationship between IAQ in schools and health. The recent review of Sundell et al. [9] looked into the available literature until 2005 and discussed five articles that have studied the school environment. They concluded from these studies that low ventilation rates are associated with increased absenteeism and more respiratory symptoms in school children, but emphasise that there is too little data available to make firm conclusions. Furthermore, they also stressed the need for more studies on the relationship between ventilation and health, especially in buildings other than offices. Since 2005, more studies on the relationship between ventilation of schools and health have been published, for example two articles relating ventilation rates in schools to illness absenteeism of the students [10,11]. Both of these studies found that lower ventilation rates are associated with higher absenteeism. Another study, on the effect of the implementation of a new ventilation system in schools, found that after installation less asthmatic symptoms were reported and exposure to airborne pollutants decreased [12].

Apart from the effects of IAQ on health, research has also focussed on the effects of ventilation on human performance. Mendell and Heath [13] reviewed the literature available until 2003 on the possible effects of poor IAQ on students’ performance and concluded that there is suggestive evidence for an association between ventilation rates and the attention and performance of students, two prerequisites of an efficient learning process. Since this review, various papers have been published regarding this topic. An observational study reported an association between classroom ventilation rates and students’ achievements on a standardised academic performance test. Based on their study the authors suggest a linear relationship between poorer classroom ventilation and lower academic achievement [14]. Four studies have used an experimental design [15-18]. Findings of these studies are inconsistent, but comparisons of the studies are difficult due to differences in study design and outcome parameters.

The levels of CO_2_ that exist indoor have long been thought to have no direct impact on occupant’s health or performance [19], but to be primarily an indicator of the level of ventilation. It has been hypothesised that the observed associations between ventilation levels and health or performance result from the fact that ventilation does not only affect the level of indoor CO_2_, but also levels of other pollutants in the indoor environment that are able to cause these adverse effects [20]. However, Satish et al. [20] conducted a laboratory experiment on the direct effects of CO_2_ at normally occurring indoor concentrations on human decision making. Their study suggests that, compared to CO_2_ concentrations of 600 ppm, at 1000 ppm and 2500 ppm a reduction in decision-making performance occurs. This may indicate the importance of considering CO_2_ in itself as an air pollutant. However, they stress that confirmation of their findings is needed.

Since there is still a need for more experimental evidence with respect to the relationship of classroom ventilation and its effect on both respiratory health and cognitive performance, the FRESH study (Forced-ventilation Related Environmental School Health) was designed. The aim of this study is to investigate whether an intervention can be used to improve classroom IAQ by increasing classroom ventilation and whether this intervention affects children’s cognitive performance and/or respiratory health. In this paper, we focus on the performance of the ventilation system in terms of achieved classroom CO_2_ concentrations.

## Methods

### Study design

The FRESH study has been designed as an intervention study with two experimental groups and one control group. Differences between the two experimental groups were created using a cross-over design. Data collection for this study took place at 17 primary schools during the heating seasons (October-April) of 2010–2011 and 2011–2012. In the first school year, ten schools participated, in the second year eight. One school participated in both the first and the second year, but with a different student population. With this exception, per school, one classroom was studied, with repeated measurements during three consecutive weeks. The first week served as baseline period, with measurements of normally existing CO_2_ levels and ventilation according to the teachers own preference. In the following two weeks, in the 12 intervention classrooms the concentrations of CO_2_ were maintained at pre-set levels of 800 and 1200 ppm, established with a mobile, custom-made mechanical ventilation device. During these weeks, the teachers were asked not to ventilate the classroom by opening doors or windows. In the six schools that acted as the control group, no intervention on ventilation took place. In these classrooms, CO_2_ levels were monitored and teachers were allowed to ventilate as they preferred.

### Participating schools

In total 18 classrooms (7^th^ grade children, ages 10–11 years) from 17 different schools were investigated in the FRESH study. These schools were all located in two regions in the north and north-eastern part of The Netherlands with comparatively low concentrations of ambient air pollutants (Zwolle and Groningen). Each region provided nine classrooms for the study. In the Zwolle region it was more difficult to find schools willing to participate, so that in the second year of the study, one school (but with a different student population) participated again. Schools were randomly selected, excluding those that were within 250 m of a busy road or highway. A total of 80 schools were asked to participate before the planned number of 18 classrooms was achieved (23% response). Many schools that did not participate in the study valued the FRESH study as important, but were too busy to take part in the (relatively invasive) FRESH study. Schools were randomly allocated to the three study arms, but were allocated to the control arm when for practical reasons it was not possible to install the ventilation system (4 schools). The exact size of the classrooms has not been measured, but classrooms in The Netherlands measure approximately 50 m^2^. The average number of students in the studied classrooms was 26, per classroom one teacher was present. All studied classrooms relied on natural ventilation through opening doors and windows to provide fresh air. Table 1 provides more information on the schools.

**Table 1:** School characteristics

**School**	**n students**	**Study region**	**Condition**	**Study period**
E1	31	Zwolle	Intervention 1 (800–1200)	Jan 2011
E2	27	Zwolle	Intervention 1 (800–1200)	March 2011
E3	22	Zwolle	Intervention 1 (800–1200)	Nov 2011
E4	30	Groningen	Intervention 1 (800–1200)	Jan 2011
E5	27	Groningen	Intervention 1 (800–1200)	Jan 2012
E6	25	Groningen	Intervention 1 (800–1200)	March 2012
E7	23	Zwolle	Intervention 2 (1200–800)	Feb 2012
E8	23	Zwolle	Intervention 2 (1200–800)	March 2012
E9	22	Zwolle	Intervention 2 (1200–800)	Jan 2012
E10	23	Groningen	Intervention 2 (1200–800)	Nov 2010
E11	29	Groningen	Intervention 2 (1200–800)	March 2011
E12	29	Groningen	Intervention 2 (1200–800)	Nov 2011
C1	36	Zwolle	Control	Oct 2010
C2	25	Zwolle	Control	Jan 2011
C3	22	Zwolle	Control	March 2011
C4	28	Groningen	Control	Jan 2011
C5	18	Groningen	Control	March 2011
C6	29	Groningen	Control	March 2012

### Intervention

**Figure 1:**
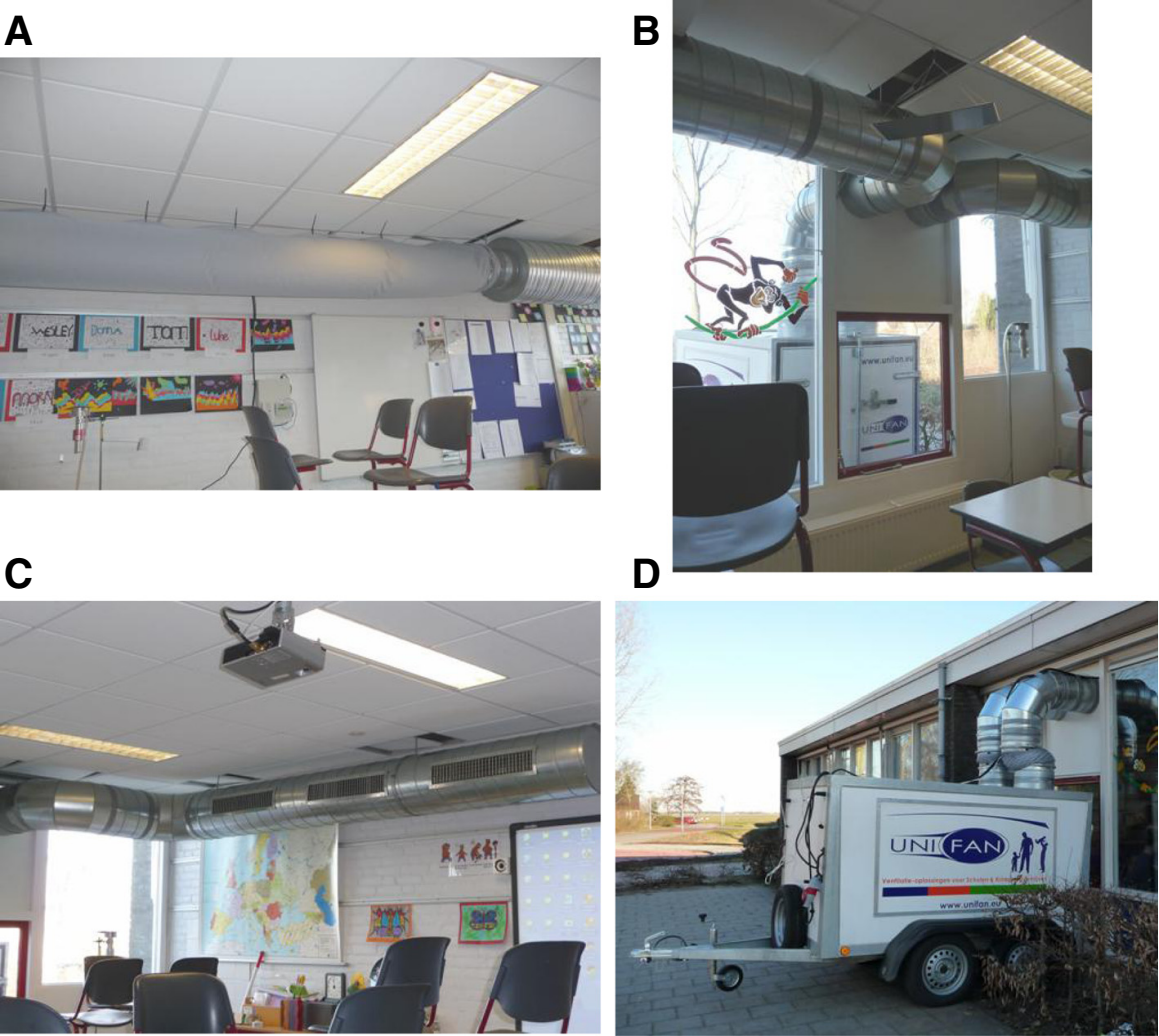
**Installation of the ventilation intervention in a classroom. A** = air sock for air supply, **B** = tailor made window pane, **C** = non-flexible duct for air exhaustion, **D** = ventilator.

In 12 schools we changed the classroom ventilation, using a specially designed and installed mechanical ventilation device. Based on a design of providing a stable ventilation flow with an adjustable outdoor air supply rate, this device consisted of an exterior constant flow fan (LAAHP12, Shandong LARK Central Air Condition Co., China) placed outdoors. Within the device outdoor air was mixed with indoor air derived from the classroom via the return system. The mixing ratio between indoor and outdoor air was depended on the setting of the targeted CO_2_ concentration and was adjusted by means of a valve in the inlet of the outdoor air supply system. The mixture of indoor and outdoor air was than heated before being introduced into the classroom with a flow of approximately 1400 m^3^/h. Simple ducting (diameter 355 mm) lead the air without filtering into the building through a tailor made window pane. In the classrooms, the air was distributed through a flexible, perforated fabric air sock. A non-flexible duct was used for air exhaust. Both the air sock and exhaust duct were attached to the ceiling of the classroom. In Figure 1 the ventilation device and the installation within a classroom are shown.

The device was CO_2_ controlled, using a real-time, self-calibrating CO_2_ sensor (Telair 6613 CO_2_ module, GE Measurement & Control, USA) to adjust the amount of outdoor air supplied, in order to achieve a target steady-state CO_2_ concentration in the classroom. This CO_2_ sensor was located at one of the walls of the classroom, at approximately 1.5 m from the floor, where possible not close to windows and doors. By means of the recirculation and constant air flow blinding of students, teachers and field investigators to the level of outdoor air supply was established. As classrooms in The Netherlands have approximately the same size, one single ventilation flow was chosen (approximately 1400 m^3^/h) that was enough to realise the targeted CO_2_ concentration without creating disturbingly high air flows within the classroom.

For this study, pre-set levels of 800 and 1200 ppm CO_2_ were defined. The lower level represents the level advised by the joint Dutch Municipal Health Services. The upper level represents the basis on which Dutch Building Regulations have formulated the minimal achievable air flow for the design of new schools [4,5]. To maintain a cross-over in the design, in half of the classrooms, we started ventilating at 800 ppm, the other six schools started with a setting of 1200 ppm. In the third week of the study, the ventilation regime changed.

To prevent thermal discomfort and create a more or less stable classroom temperature, the device was equipped with an air pump able to both heat and cool the outdoor air before it was introduced into the classrooms. Classroom temperature was set at 21°C, to minimize differences between the schools. Based on measurements of a real-time temperature sensor (located at the same position as the CO_2_ sensor) cooling or heating of the supplied air was adjusted according to the classroom temperature. As the experiment was carried out in winter seasons, classroom temperature was higher than outdoor temperature. Even though no measurements were performed of the exact temperature of supplied air, it is to be expected that this air was heated. When classroom temperature exceeded 21°C, colder air was supplied to lower the indoor temperature. Furthermore, the system was designed to maintain system noise below 35 dB (A). This value has shown to be the threshold for annoyance and disturbance [21].

### Indoor measurements

During the study weeks, each classroom was equipped with two data loggers (GRP-300 Pro (ATAL, The Netherlands) in study region 1 and ATV-IAQ set (ATAL, The Netherlands) in study region 2) for CO_2_, temperature and relative humidity. These data loggers were calibrated each year by the manufacturer. The loggers were positioned as much as possible at the height of the desks of the pupils and on the opposite sides of the classroom. Log interval was 4 minutes. From the two data loggers the average was taken to represent classroom CO_2_, temperature and relative humidity. All data reported in this paper are restricted to periods of actual classroom occupation excluding breaks and periods when students were elsewhere (e.g. gym).

### Outdoor measurements

The selected schools were not located to obvious sources of CO_2_, therefore no continuous measurements of outdoor CO_2_ concentrations were performed. To get an indication of outdoor CO_2_ concentrations, short time frame measurements of approximately 5 minutes were performed just outside the school building, using the same type of CO_2_ data loggers that were used for indoor measurements. Measurements took place at the beginning and end of each week. Data on 24 h-average outdoor temperature and relative humidity were obtained from the two weather stations (Eelde and Hoogeveen) of the Royal Netherlands Meteorological Institute closest to the study regions.

### Ethical approval

The study design and protocols have approved by the ‘Central Committee on Research involving Human Subjects’ (CCMO, The Hague) on February 23, 2010 and is registered under number 120620026.

### Statistical analysis

The data were analysed using PASW Statistics 18 and SAS 9.2. Significance was tested against an α = 0.05. The effect of the intervention, as well as differences between the two settings of the intervention (800 ppm and 1200 ppm) were tested by means of mixed models with random school intercepts to take into account the dependency of the repeated measurements performed in the same classrooms.

## Results

Mean indoor CO_2_ concentrations, temperature and relative humidity during classroom occupation per school per week are presented in Tables 2, 3 and 4.

**Table 2:** **Mean indoor CO**_**2 **_**concentration (ppm) per school per week**

		**Week 1**				**Week 2**				**Week 3**			
**School**	**Condition**	**n**	**mean**	**sd**	**P98**	**n**	**mean**	**Sd**	**P98**	**n**	**mean**	**sd**	**P98**
E1	Intervention 1 (800–1200)	344	1365	531	2991	344	902	85	1128	280	899	83	1051
E2		352	1337	460	2351	345	802	74	903	294	908	158	1126
E3		286	1143	398	2052	285	753	78	902	320	900	99	1085
E4		215	1648	353	2395	312	843	48	930	350	1059	99	1203
E5		347	1466	330	2322	295	906	147	1347	257	1063	134	1276
E6		255	2000	602	3321	270	915	44	993	297	1077	96	1195
E7	Intervention 2 (1200–800)	356	1323	291	1963	356	993	140	1221	356	820	85	937
E8		354	1049	158	1313	321	905	130	1124	353	743	87	887
E9		265	1763	423	2507	301	975	84	1159	336	764	51	853
E10		222	763	131	1153	272	887	119	1150	334	858	76	938
E11		367	1762	625	3064	352	995	151	1168	336	858	105	1018
E12		347	1171	213	1553	343	1034	108	1182	309	925	70	1045
C1	Control	380	1393	483	2446	379	2137	591	3179	379	2328	483	3197
C2		342	1176	289	1694	342	1100	255	1523	342	1249	347	1838
C3		351	1112	333	1789	335	1132	304	1827	327	996	240	1527
C4		350	1389	425	2264	340	1274	357	1982	340	1191	389	2362
C5		353	779	177	1166	328	864	159	1234	312	740	151	1098
C6		282	1399	311	1975	344	1677	377	2318	336	1509	538	2879

**Table 3:** Mean indoor temperature (°C) per school per week

		**Week 1**			**Week 2**			**Week 3**		
**School**	**Condition**	**n**	**mean**	**sd**	**n**	**mean**	**sd**	**n**	**mean**	**sd**
E1	Intervention 1 (800–1200)	344	20.2	1.4	344	20.3	0.9	280	20.3	0.9
E2		352	21.1	1.1	345	19.1	0.8	294	19.1	0.8
E3		286	21.0	0.9	285	19.3	1.7	320	19.3	1.7
E4		215	19.2	1.1	312	18.5	1.2	350	18.5	1.2
E5		347	21.2	1.1	295	21.4	1.5	257	21.4	1.5
E6		255	20.4	1.0	270	20.2	0.5	297	20.2	0.5
E7	Intervention 2 (1200–800)	356	21.6	0.9	356	20.4	1.0	356	20.4	1.0
E8		354	19.2	0.8	321	19.7	1.2	353	19.7	1.2
E9		265	21.8	1.5	301	20.8	1.9	336	20.8	1.9
E10		222	23.0	0.8	272	23.3	0.9	334	23.3	0.9
E11		367	20.9	1.1	352	20.8	1.3	336	20.8	1.3
E12		347	22.0	1.3	343	21.1	0.8	309	21.1	0.8
C1	Control	380	22.6	1.2	379	20.6	1.5	379	20.6	1.5
C2		342	21.2	0.8	342	20.7	0.6	342	20.7	0.6
C3		351	19.8	1.3	335	20.1	1.6	327	20.1	1.6
C4		350	20.9	0.6	340	21.8	0.8	340	21.8	0.8
C5		353	20.4	1.2	328	22.3	0.6	312	22.3	0.6
C6		282	19.7	1.0	344	20.1	1.0	336	20.1	1.0

**Table 4:** Mean indoor relative humidity (%) per school per week

		**Week 1**			**Week 2**			**Week 3**		
**School**	**Condition**	**n**	**mean**	**sd**	**n**	**mean**	**sd**	**n**	**mean**	**sd**
E1	Intervention 1 (800–1200)	344	33.6	6.4	344	38.0	11.7	280	30.3	4.0
E2		352	30.4	6.2	345	28.3	10.4	294	44.9	6.8
E3		286	54.4	5.6	285	41.5	7.6	320	32.0	8.6
E4		215	45.5	7.8	312	35.3	6.8	350	37.3	4.2
E5		347	49.8	6.2	295	30.7	4.0	257	32.7	3.2
E6		255	48.2	3.4	270	40.2	3.4	297	42.1	4.4
E7	Intervention 2 (1200–800)	356	40.1	5.6	356	27.9	4.6	356	29.2	3.0
E8		354	42.7	3.9	321	42.4	3.7	353	40.4	3.0
E9		265	27.1	4.4	301	15.7	3.0	336	30.2	2.9
E10		222	48.7	4.0	272	31.4	2.9	334	32.6	1.6
E11		367	46.6	7.9	352	31.4	7.3	336	39.9	7.5
E12		347	53.8	4.7	343	41.9	4.8	309	33.0	5.9
C1	Control	380	62.3	2.9	379	54.6	4.5	379	55.0	2.2
C2		342	33.4	6.5	342	32.7	6.3	342	37.3	4.2
C3		351	42.3	4.3	335	43.7	6.5	327	47.0	6.3
C4		350	37.1	4.4	340	39.6	7.6	340	36.0	5.7
C5		353	35.9	4.1	328	35.8	6.9	312	40.1	5.3
C6		282	27.6	4.2	344	29.4	4.2	336	42.8	4.0

During the first week (baseline) mean classroom CO_2_ concentration was 1335 ppm (sd = 325) with a range of 763–2000 ppm. In the classrooms allocated to become intervention schools, mean CO_2_ concentration was 1399 ppm (sd = 350), the control classrooms had an average CO_2_ concentration of 1208 ppm (sd = 244). Only two classrooms (E10 and C5) had mean CO_2_ concentration lower than 800 ppm at baseline, and another five classrooms had mean CO_2_ concentrations lower than 1200 ppm.

**Figure 2:**
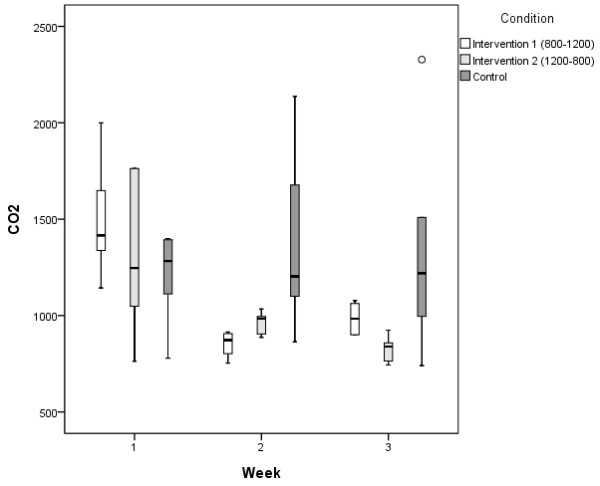
**Boxplot of mean CO**_**2 **_**concentration per condition per week.**

In the second week, we started the intervention in 12 classrooms. In those 12 classrooms, on average we decreased mean CO_2_ with 491 ppm compared to baseline (sd = 324, range: -1085–124 ppm). With the setting of the ventilation set at 800 ppm, the average CO_2_ concentration was 841 ppm (sd = 65) with a range of 743–925 ppm. When set at 1200 ppm, the average CO_2_ concentration was 975 ppm (sd = 73, range: 887–1077 ppm). In the control classrooms, during the second and third week, CO_2_ concentrations ranged from 740 to 2328 ppm, with an average mean CO_2_ concentration of 1350 ppm (sd = 486). Figure 2 displays the boxplot of CO_2_ concentrations per condition per week. The P98 results confirm that the ventilation device was able to maintain a maximum level of 1200 ppm CO_2_, whereas it was more difficult to keep CO_2_ levels below 800 ppm (Table 2).

Table 5 provides the results from our mixed model analysis. From this analysis we can conclude that classroom CO_2_ levels were statistically significantly decreased during the intervention (F (2,10) = 17.59, p < 0.001). Compared to baseline, the estimated mean decrease in CO_2_ with the setting at 800 ppm was 558 ppm (SE = 97.8). For the setting of 1200 ppm, the estimated mean decrease was 424 ppm (SE = 97.8). The mean difference in decrease compared to baseline between the two settings of the intervention was 134 ppm (SE = 29.3, t (10) = 4.57, p = 0.001).

**Table 5:** **Mean decrease of CO**_**2 **_**(ppm), temperature (°C) or relative humidity (%) compared to baseline measurements**

	**CO**_**2 **_**(ppm)**			**T (°C)**			**RH (%)**		
**Setting**	**mean decrease**	**SE**	**p**	**mean decrease**	**SE**	**p**	**mean decrease**	**SE**	**p**
800 ppm	558	97.8	<0.001	0.56	0.35	0.144	8.5	2.4	0.005
1200 ppm	424	97.8	0.002	0.10	0.35	0.784	9.3	2.4	0.003
Difference between 800 and 1200 ppm	134	29.3	0.001	0.46	0.24	0.088	0.8	2.3	0.734

**Figure 3:**
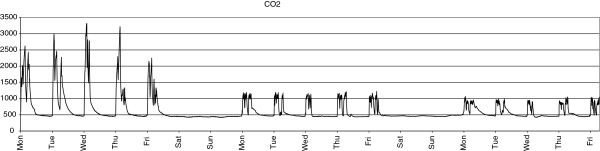
**Three week graph of mean CO**_**2 **_**concentration in one of the experimental schools.**

The result of implementation of the ventilation intervention and its effect on the CO_2_ in a classroom is illustrated in Figure 3. This graph displays the CO_2_ concentration during the three weeks of the study in one of the experimental classrooms. The graph shows how in the first week, high CO_2_ peak concentrations exist, which no longer occur during the second and third week. Also, it shows how the CO_2_ concentrations are much more stable in the two intervention weeks. Furthermore, the graph shows the (slight) difference in CO_2_ concentration during the second (ventilation set at 1200 ppm) and third (800 ppm) week.

The intervention was designed in such way that classroom temperature did not decrease as a result of supplying (cold) outdoor air. At baseline, average indoor temperature was 20.9°C (sd = 1.1, range: 19.2–23.0°C). In the intervention classrooms average temperature during weeks two and three was 20.6°C (sd = 1.0, range: 18.5–23.3°C), in the control classrooms average temperature was 20.9°C (sd = 1.2, range: 18.5–22.5°C). No significant effect of the intervention on classroom temperature was found (F (2,10) = 2.13, p = 0.170), nor on differences between the two intervention settings (Table 5).

Indoor relative humidity at baseline was 42.2% (sd = 9.9, range: 27.1–62.3%), in weeks two and three average relative humidity was 41.2% (sd = 8.0, range: 29.4–55.0%) in the control classrooms and 34.5% (sd = 6.6, range: 16.7–44.9%) in the intervention classrooms. This decrease in relative humidity due to the intervention appeared to be statistically significant (F (2,10) = 4.16, p = 0.049). No significant difference between the two intervention conditions was found (Table 5).

During the study, outdoor CO_2_ concentration was on average 471 ppm (sd = 53, range: 350–660 ppm), mean outdoor temperature was 4.7°C (sd = 5.1, range: -12.7–16.9°C), and mean outdoor relative humidity was 87.1% (sd = 8.5, range: 54–100%).

## Discussion

This study showed that it is possible to use a portable, tailor made mechanical ventilation device to improve outdoor air supply in schools during the heating season. In the classrooms where we intervened we found an average decrease of 491 ppm CO_2_ with, however, little difference between the two experimental conditions. The target value of 1200 ppm was more than met, however the target value of 800 ppm proved to be more difficult to achieve. To what extent this is due to differences in CO_2_ concentrations measured at the location of the system sensor and the location of our two data loggers we do not know as the system sensor was unable to log the CO_2_ concentrations, nor was it equipped with a display enabling us to read measured CO_2_ concentrations by the system sensor. Another possible explanation could be that the ventilation device appeared to have not enough capacity to lower CO_2_ concentration to 800 ppm during classroom occupation. Technical specifications suggest that this should not have been the case, however, we did not measure true air displacement of our installation in the field as we focussed our study design on obtaining specific indoor CO_2_ concentrations rather than on achieving specific ventilation rates.

In all but one classroom, the intervention was able to decrease CO_2_ concentration. The level of decrease varied per classroom, as this is related to CO_2_ concentration measured at baseline. The highest decrease in CO_2_ concentration was observed in school E6, where we lowered mean CO_2_ concentration from 2000 ppm to 915 ppm. In one school CO_2_ levels slightly increased after implementation of the intervention (school E10), this however was due to the high ventilation rate in the baseline week which produced low CO_2_ concentrations that we did not need to lower further. In seven schools we found baseline CO_2_ concentrations lower than 1200 ppm, in two schools the average CO_2_ concentration in the first week was lower than 800 ppm. This number is higher that we had expected based on the studies from 2007 [2,3]. It is plausible that since 2007 ventilation behaviour in schools has improved. The study by Versteeg [2] resulted in media-attention and a political debate in the Dutch government. Moreover, it could well be that the participation in the FRESH study directly influenced the teachers’ (and pupils’) awareness of the importance of proper classroom ventilation, resulting in relatively low baseline CO_2_ concentrations. The decreased relative humidity indoors during the intervention period may be explained by differences in outdoor and indoor temperature between baseline and intervention periods. Especially in cold climates, low indoor relative humidity is associated with increased ventilation rates [7].

Recently various other classroom ventilation intervention studies have been published, most of them predefined a contrast aimed to be achieved by the intervention. One of these studies, by Twardella et al. [16] adjusted the mechanical ventilation within 20 classrooms of six schools. They either up- or down-regulated the ventilation to achieve CO_2_ levels of < 1000 ppm (‘better than usual’) or CO_2_ concentrations of 2000 to 2500 ppm (‘worse than usual’). Each condition was implemented for 2 days. They report that it was difficult to regulate the ventilation in such way that the targeted CO_2_ levels were achieved: only on half of the days of the ‘worse than usual’ condition CO_2_ concentrations were higher than 2000 ppm and on 22 (of the 40) days of the ‘better than usual’ condition CO_2_ concentrations were below 1000 ppm. Wargocki and Wyon [22] performed three experiments in which they also adjusted the existing outdoor air supply of the mechanical ventilation of schools by altering the fan capacity. They aimed on increasing ventilation rates from approximately 3 to 10 L/s per person. Using a general mass balance equation from measured CO_2_ concentrations, they were able to estimate the actual effective ventilation rates. In the first experiment estimated mean effective ventilation rates were 4 L/s and 8.5 L/s per person, in the second experiment these ventilation rates were 3 L/s and 6.5 L/s per person and in the third experiment 5 L/s and 9.5 L/s per person. This shows that while they aimed for a threefold increase of the ventilation rates, the estimated actual effective ventilation rates were doubled. Bakó-Biró et al. [15] intervened upon classroom ventilation using an installation similar to the one we used in the FRESH study. The biggest difference with our study is that they did not adjust ventilation to achieve predefined levels of CO_2_, but used the installation to either supply fresh air or recirculate the indoor air in a blinded fashion. As this study aimed at comparing high and low levels of outdoor air supply, with their intervention set at recirculation, they were able to achieve big differences in CO_2_ concentration between the two experimental conditions. In their study, Smedje and Norbäck were able to study the change in indoor air quality in schools that renewed their ventilation system [12]. They observed that air exchange rates improved, and that associated CO_2_ concentrations decreased on average by 270 ppm due to a new ventilation system. Furthermore, they also reported a significant decrease in relative humidity in schools with a new ventilation system (-10%), compared to schools that did not change their ventilation system (-2%).

## Conclusions

Various studies, including our own, show that intervening on classroom ventilation is effective if one wants to change indoor CO_2_ concentrations. Furthermore, both our own study and the studies of Twardella et al. [16] and Wargocki and Wyon [22] show that field experiments are not comparable with laboratory experiments and that it can be challenging to execute the study as designed. Altogether, our study has shown that classrooms CO_2_ levels can be significantly reduced by installing a CO_2_ controlled mechanical ventilation system.

## Abbreviations

IAQ: Indoor air quality; ppm: Parts per million; FRESH: Forced-ventilation related environmental school health (acronym for the study); m3/h: Cubic meters per hour; dB(A): A-weighted decibels; L/s: Liters per second; sd: Standard deviation; SE: Standard error.

## Competing interests

The authors declare that they have no competing interests.

## Author’s contributions

JTMR supported in the design of the study, led and participated in field work in one of the study regions, conducted data processing, analysis, and interpretation and drafted the manuscript. MV contributed to the study and intervention design, participated in data collection in one of the study regions and managed the overall project. MV furthermore helped draft the manuscript. JTvG contributed to the intervention design, participated in data collection in one of the study regions and reviewed the finalised manuscript. UG contributed to the statistical analysis and interpretation of the data and critically reviewed the manuscript. FD and BB conceived of and managed the project and helped prepare and critically review the draft. All authors have read and approved the final manuscript.
